# Assessing the effectiveness of a community health advisor plus screen to save educational intervention on stool-based testing adherence in an African American safety net clinic population: study protocol for a randomized pragmatic trial

**DOI:** 10.1186/s13063-022-06076-4

**Published:** 2022-02-15

**Authors:** John S. Luque, Olayemi O. Matthew, Deloria R. Jackson, Matthew A. Vargas, Tifini Austin, Askal Ali, Gebre E. Kiros, Cynthia M. Harris, Rima Tawk, Clement K. Gwede, Kristin Wallace, Pascal Jean-Pierre

**Affiliations:** 1 College of Pharmacy & Pharmaceutical Sciences, Institute of Public Health, Florida A&M University, 1515 South Martin Luther King Jr. Blvd., Tallahassee, FL 32307 USA; 2 Division of Population Sciences, Department of Health Outcomes and Behavior, Moffitt Cancer Center, Tampa, FL 33612 USA; 3 Department of Public Health Sciences, College of Medicine, Medical University of South Carolina, 68 President Street, Charleston, SC 29425 USA; 4 Hollings Cancer Center, Medical University of South Carolina, 86 Jonathan Lucas Street, Charleston, SC 29425 USA; 5 Department of Behavioral Sciences and Social Medicine, College of Medicine, Florida State University, 1115 West Call Street, Tallahassee, FL 32306 USA

**Keywords:** Colorectal cancer screening, Randomized controlled trial, Screening, Cancer prevention, African Americans

## Abstract

**Background:**

Colorectal cancer (CRC) is the second most deadly cancer affecting US adults and is also one of the most treatable cancers when detected at an earlier clinical stage of disease through screening. CRC health disparities experienced by African Americans are due in part to the later stage of diagnosis, suggesting the importance of improving African Americans’ CRC screening participation. The national Screen to Save (S2S) initiative employs a community health educator to deliver CRC screening education which can be tailored for specific populations, and such approaches have increased CRC screening rates in disadvantaged and racial/ethnic minority populations.

**Methods/design:**

In this trial emphasizing stool-based CRC screening, focus groups informed the development of an adapted S2S video and brochure tailored for African Americans and identified preferred motivational text messages for a multicomponent community health advisor (CHA) intervention. A CHA hired from the community was trained to deliver a 6-week CRC educational intervention consisting of an initial face-to-face meeting followed by 5 weeks of calls and texts. Interested eligible persons are enrolled primarily through recruitment by two partnering community health centers (CHCs) and secondarily through various outreach channels and, after consenting and completing a baseline survey, are randomly assigned to one of two study arms. The CHCs are blinded to study arm assignment. Intervention arm participants receive the brochure and CHA intervention while participants assigned to the control group receive only the brochure. All participants receive a stool-based CRC screening test from their health center, and the primary outcome is the completion of the screening test at 12 months. Secondary objectives are to estimate the effect of the intervention on mediating factors, explore the effect of moderating factors, and perform a cost-effectiveness analysis of the CHA intervention.

**Discussion:**

The TUNE-UP study will enhance understanding about CRC screening in African Americans obtaining primary health care through CHCs and is one of the very few studies to examine a CHA intervention in this context. A better understanding of the mechanisms by which the intervention affects patient beliefs and behaviors will help focus future research while the exploratory cost-effectiveness analysis will inform CHCs’ decision-making about implementing a CHA program to increase screening and reduce cancer health disparities.

**Trial registration:**

ClinicalTrials.govNCT04304001. Registered on March 11, 2020.

## Administrative information

Trial Sponsor: Florida A&M University, Charles Weatherford, PhD, Vice President for Research, Office of the Vice President, Division of Research, 410 Foote-Hilyer Administration Center, Tallahassee, FL 32307-3200.

The sponsor principal investigator (JL) has authority over research activities, writing of reports, and submission of written reports.

Principal investigator: Design and conduct of TUNE-UP, Preparation of protocol and revisions, publication of study reports. All lead investigators are steering committee members involved with overseeing the trial and meet weekly. Data verification, randomization, data management is led by the study biostatistician (GK). The study coordinator (MV) is responsible for identifying potential research participants in partnership with the Community Health Centers, who send out recruitment messages to contact the study coordinator.

## Background

### Colorectal cancer rates and screening in African Americans

Colorectal cancer (CRC) is the second most common type of cancer and second leading cause of cancer-related deaths in the USA affecting both men and women, after lung cancer. It is projected that close to 53,000 people in the USA will die of CRC in 2021 [1]. CRC is also one of the most treatable forms of cancer when detected at an earlier stage of disease. Through regular screening, cancers can be identified at earlier stages of carcinogenesis and in some patients may allow removal of precancerous lesions which prevent CRC from developing.

African Americans have the highest rates of CRC compared to any other racial or ethnic group in the USA [2]. The disproportionate burden of CRC incidence and mortality rates among African Americans compared to their white counterparts has persisted despite consistent overall declines across other racial and ethnic groups. Compared to whites, the CRC mortality rate was approximately 47% higher for African American men and 34% higher for African American women [2]. The 5-year CRC survival rate from 2010 to 2016 was 59% for African Americans compared to 65% for whites [1]. African American CRC patients present with a more advanced stage of disease at diagnosis compared to white patients which highlights the importance of increased screening participation, since localized disease has a 90% 5-year relative survival rate [1].

According to the CDC, approximately 69% of the US population 50–75 years were reported to be up to date with CRC screening in 2018, with the largest variations in screening observed for segments of the population without health insurance, from racial/ethnic minority groups, those living in rural areas, and those living in areas with low availability of gastroenterologists who can perform colonoscopies [3]. The causes of black-white CRC disparities are multifactorial and include environmental, sociocultural, and genetic factors [4]. For example, some factors helping to explain CRC disparities in mortality include disparate levels of obesity and physical activity, diet, smoking rates, environmental exposures, and access to health care that affect the level and quality of screening, diagnosis, and treatment [4]. One evidence-based approach to improve CRC screening participation is to culturally tailor interventions as part of a patient navigation or community outreach strategy to regularly inform and educate medically underserved communities on the importance of participating in recommended CRC screenings, whatever the actual screening method [5].

### CRC screening recommendations

Beginning over two decades ago, overall CRC incidence rates started declining steadily because of increased uptake of colonoscopy screening; however, rates have continued to increase in the <65-year-old population [1]. Because of the rise in early-onset CRC—before 50 years old—the recommended age for an average-risk patient to begin CRC screening was recently changed to 45 years from 50 years. An assessment of the magnitude of perceived net benefits issued by the US Preventive Services Task Force (USPSTF) provides an updated B recommendation for all average-risk persons to participate in CRC screening between the ages of 45 and 49 years, while maintaining an A recommendation for adults between the ages of 50 and 75 years. This recommendation update will allow earlier CRC screening to be covered by health insurance without a co-pay for routine screening [6]. The American Cancer Society had previously released the same recommendation for earlier routine CRC screening in 2018 [7].

Access and timely use of appropriate screening procedures are of utmost importance in bridging the disparities in CRC incidence, mortality, and 5-year survival rates for African Americans. Recommended CRC screening methods range from clinic-based to home-based tests. The gold standard CRC screening test is colonoscopy where the entire large bowel is visualized but requires colonic preparation and sedation and is often, but not always readily accessible in some locations, especially in rural communities. Although colonoscopy screening has the advantage of removing preinvasive lesions before they become cancer, and a longer screening interval if no pathology is identified (up to 10 years), average-risk patients may prefer a less invasive option. The stool-based tests are less costly and easier to access and complete. To boost acceptance and screening rates among medically underserved populations, several studies have stressed the positive impact of annual stool-based tests [8, 9]. One potential issue however is that follow-up colonoscopy after a positive stool-based test is considered a diagnostic exam, which may incur higher costs depending on one’s health insurance coverage and present another potential barrier to prevention [10].

### Stool-based testing approaches

There are three common recommended stool-based screening methods [7, 11]. The guaiac-based fecal blood test or gFOBT tests chemicals within the guaiac to detect if there is any blood in the stool [6]. With this test, patients receive testing kits from their health care provider to perform a self-test at home. The assessment includes using a stick or brush to gather a small amount of stool for testing. The kit is then returned to the provider where the stool sample is tested for any presence of blood. This test is usually performed once a year [6]. A second option for stool-based testing is the fecal immunochemical test (FIT) which uses antibodies to detect blood in the stool [6]. Like the gFOBT test, patients are given a kit to take home to obtain a stool sample and send to a laboratory for testing. The FIT is preferable over the gFOBT because it is a specific marker of human blood and dietary restrictions are not needed and is also done annually [6]. The third option is the multi-target stool DNA test, which is the only stool DNA test approved by the US Food and Drug Administration [6]. For this test, the stool sample consists of an entire bowel movement that is sent by and returned to the company directly and includes a FIT component and DNA biomarkers for neoplastic cells that shed in the lining of the colon and rectum [6]. This test is usually performed less frequently, once every 3 years, and may have higher sensitivity than the other stool-based tests [6, 12]. For the purposes of this trial, the FIT is provided to research participants by their community health center (CHC).

### Community health advisor and patient education materials

A community health advisor (CHA) is a member of the community who has been trained to deliver health education and other services to assist community members to engage in healthy behaviors such as cancer screening, diabetes management, or nutrition counseling. Other positions who fill similar roles as the CHA include community health workers, *promotoras*, lay health advisors, and patient navigators. These individuals are trusted community members operating in a culturally competent manner who are either paid employees or work as volunteers to connect members of the community with health services. The use of a patient navigator or similar advocate not only helps to increase cancer screenings in hard-to-reach populations but is also cost-effective [13]. The use of CHAs in cancer prevention in a CHC patient population can be an effective intervention strategy to both recruit individuals to stool-based CRC screening and navigate to follow-up colonoscopy screening using safety net referral networks, and the comparator used in many of these types of screening outcome studies is usual care [14, 15].

Screen to Save (S2S) is an initiative of the National Cancer Institute (NCI) to increase CRC screening in the USA [16]. One component of S2S is an educational PowerPoint presentation which may be tailored depending on the priority population of interest. The educational presentation component describes CRC incidence and mortality, risk factors, lifestyle changes, CRC screening options (stool-based tests, sigmoidoscopy, colonoscopy, CT colonography), and current CRC screening recommendations. The NCI initiative employs a community health educator working in an NCI-designated cancer center to deliver the S2S program either in-person or at community outreach events as an evidence-based approach for increasing CRC screening. Evaluation findings of the S2S national initiative reported positive outcomes in terms of CRC knowledge and screening participation following attendance at outreach events where the S2S materials were delivered [17]. A study in Ohio using the S2S intervention with African Americans and Appalachian whites reported that although the S2S educational intervention was successful in increasing CRC knowledge, this marginal gain in knowledge did not translate into increases in intention to receive CRC screening [18]. The study recommended focus group research to improve the components of the PowerPoint presentation, which was reported to be more effective in increasing knowledge than the inflatable colon [18]. The combination of a tailored S2S education intervention with the CHA education outreach represents a multicomponent approach to address challenges in completing CRC screening for African Americans in a safety net health setting. In this study, by partnering academic researchers with CHC personnel to deliver CRC education and screening outreach, the desired outcome of increased screening is potentially improved.

### Conceptual framework

This study examines decision-making factors that lead African Americans to participate in CRC screening. Such factors include perceived susceptibility, perceived benefits, perceived barriers, fear and fatalistic beliefs, social norms, self-efficacy, cultural factors, and knowledge. Hypothesized moderating variables which may influence the strength of the relationship between receiving the intervention and CRC screening adherence include a family history of cancer and health literacy. These variables included in our conceptual framework are drawn from health behavior theories including Social Cognitive Theory, the Health Belief Model, and the Theory of Planned Behavior/Theory of Reasoned Action [19–21]. The study measures the effect of the CHA intervention to increase knowledge about CRC and CRC screening, perceived susceptibility, and self-efficacy and to decrease fear and fatalistic beliefs which is hypothesized to result in higher CRC screening test completion.

## Study objectives

1. To determine the effectiveness of a CHA intervention, which includes patient education videos and a tailored brochure, relative to a control group receiving only a tailored brochure, on completion of stool-based CRC screening (completing the FIT by 12 months post-randomization) in African Americans who are patients of participating CHCs and not up to date with screening
2. To estimate the effect of a CHA intervention on mediators (knowledge, perceived susceptibility, self-efficacy, and fear and fatalism) associated with completion of stool-based CRC screening
3. To explore moderators (family history of cancer and health literacy) between the CHA intervention and completion of stool-based CRC screening
4. To conduct a cost-effectiveness analysis of the CHA intervention

The primary hypothesis is that participants receiving the CHA intervention will exhibit higher rates of stool-based CRC screening completion than participants in the control group, because participants in the intervention group will have higher levels of knowledge, perceived susceptibility, and self-efficacy and lower levels of fear and fatalism after receiving the CHA intervention.

## Methods/design

### Overview of the study design

**Fig. 1:**
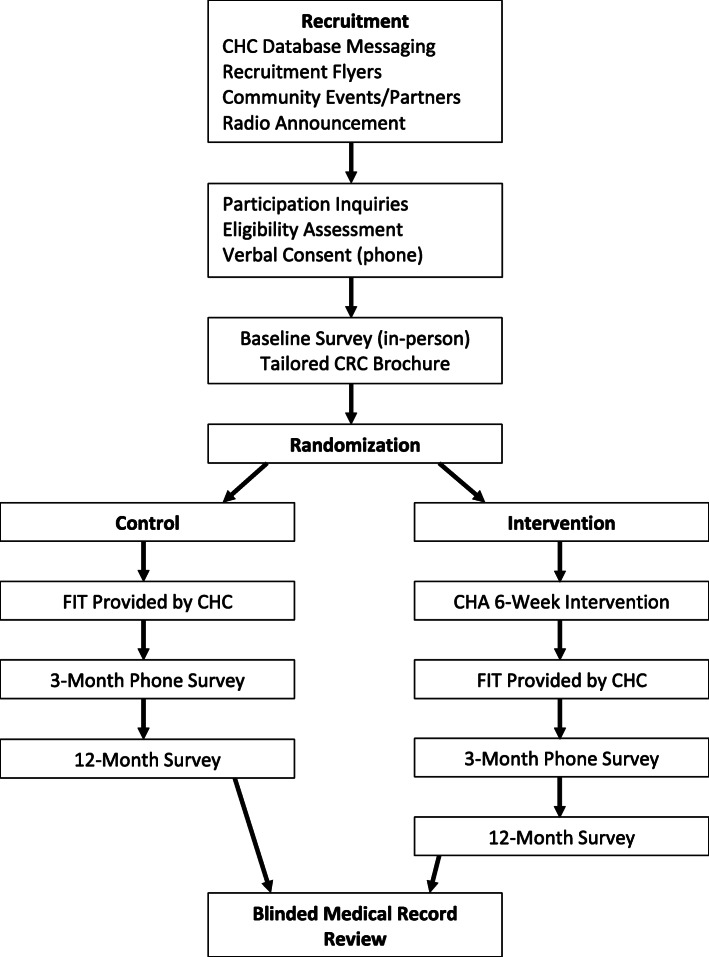
Flowchart of the intervention trial

The TUNE-UP study is designed as a two-group pretest/posttest pragmatic randomized controlled trial. The study uses the parallel group, two-arm superiority trial design with a 1:1 allocation ratio. The pragmatic trial aims to test the effectiveness of the intervention in a generalizable setting. To conduct the intervention trial, 244 participants will be randomized at the time of enrollment following completion of the baseline survey (Fig. 1). The two experimental arms are (1) an intervention group which receives the adapted S2S educational materials, a tailored brochure for African Americans on CRC screening with input from focus group research, and CHA in-person and cellphone education and counseling supplemented by text messages and (2) a control group which receives the brochure only. Participants in both study arms receive communication from their CHC to receive their stool-based testing kit. The primary outcome is CRC screening by 12 months post-intervention (i.e., receipt of the stool-based test). The intermediary CRC screening outcome is measured at 3 months. Secondary outcomes also include the intention to receive CRC screening or actual receipt of CRC screening at 3 months post-intervention.

### Study participants and setting

African American men and women aged 45 to 64 years are included in the study. We selected the age range because of three factors: (1) CRC screening rates increased from 58 to 68% in the 65-year and older cohort, possibly due to Medicare coverage; (2) an increasing number of CRC cases are diagnosed in people under 50 years and ACS and USPSTF recently updated their recommendations to include adults 45 years and older as persons with “average risk” and recommended for screening; and (3) the use of technologies such as text messaging is more likely to be familiar to participants under 65 years [11]. Participants are screened for eligibility after contacting the project coordinator according to the following inclusion criteria: (1) 45–64 years of age, (2) self-identify as African American, (3) have a working cellphone, (4) resident of Florida, and (5) not up to date with CRC screening per established screening guidelines (i.e., no stool tests in > 9 months, no colonoscopy within 9 years, and no flexible sigmoidoscopy within 4 years) [11]. Exclusion criteria are previous history of CRC, precancerous colorectal polyps, or co-morbid conditions, such as inflammatory bowel disease or Crohn’s disease. The project coordinator reads the consent form and obtains verbal consent from eligible persons interested in participating in the trial. Participants receive the consent form signed by the project coordinator with complete information on the study and university contact information when meeting to complete the baseline survey. Based on recruitment and outreach, research participants are patients of one of two community health centers in the greater Tallahassee, Florida area, and primarily reside in two North Florida counties: Leon or Gadsden.

### Randomization and blinding

The study uses the block randomization method. This method is commonly used in clinical trial designs to lower bias and attain balance in the assignment of study participants to treatment and control groups. The trial randomizes participants at the individual level, stratified by the two CHC sites, using a 1:1 ratio between intervention and control arms. The randomization algorithm uses randomly permuted blocks with random block sizes for the purpose of assigning participants. The random selection of block sizes has the advantage of reducing selection bias. The randomization file was produced in SAS v. 9.4 by the biostatistician. The principal investigator informs the study coordinator of the study arm assignment using ID numbers after the patient gives consent to participate in the study and completes the baseline survey. If a participant is assigned to the intervention arm, the study coordinator informs the CHA so the participant can be contacted to complete the intervention arm protocol.

### Intervention and control groups

All participants receive the tailored educational brochure at the initial visit when they complete the baseline survey with the project coordinator. The participants who are randomized to the intervention group view the S2S video, and the CHA explains the contents if there are questions. This is followed by 2 weeks of short CHA follow-up calls, concluding with 3 weeks of motivational text messages on CRC screening. Participants who are randomized to the control group receive the educational brochure only. Both intervention and control groups receive the FIT from their CHC either in the mail, in person, or during a scheduled patient visit. The CHCs are blinded to the study arm. There are no circumstances where unblinding participants for the CHCs would be necessary given all participants receive the FIT from their CHC.

### Community health advisor and educational materials

In the context of the behavioral clinical trial, a CHA was trained by the research team to deliver the 6-week intervention consisting of an initial face-to-face CRC educational presentation using the S2S video, 2 weeks of phone-call follow-up, and 3 weeks of text message follow-up. The intervention’s one-on-one education, small media, follow-up reminders, and reduction of structural barriers to screening align with client-oriented recommendations of the Community Guide to Preventive Services [22]. In alignment with the NCI’s goal of increasing CRC screening rates, our study, Test Up Now Education Program (TUNE-UP), created and adapted materials which consist of an educational brochure and a narrated presentation video—based on the S2S materials—to deliver a culturally tailored intervention to African American participants. The intervention is delivered by the CHA to African Americans in North Florida who are patients of the participating CHCs and are not up to date with CRC screening.

The adapted S2S PowerPoint developed by the research team was professionally produced into an approximately 11-min video narrated by an African American woman. As with the educational brochure, the adapted S2S video featured culturally tailored content including anatomical figures with brown skin tone and a photo of a local African American couple, a pastor and his wife, who are well-known in the community. After the participant views the adapted S2S video on the CHA’s tablet, the CHA asks if the participant wishes to discuss the video or ask any questions. After allowing 2–3 min for discussion, the CHA shows the participant a 5-min FIT patient education video. This video walks the viewer through the steps of completing their at-home screening test. The video was obtained from the manufacturer of the FIT after the research team was informed by CHCs that it was the primary FIT they use. Once the participant has finished viewing the second video, the CHA again engages the participant to discuss any topics or questions the participant may have. The CHA uses a fidelity checklist to document the completion of the week 1 educational presentation and make appropriate notes. In weeks 2 and 3 of the intervention, the CHA calls the participant to ascertain receipt of the FIT from their CHC and the participant’s completion of their FIT. Fidelity checklist phone scripts allow the CHA to document the delivery of the calls and facilitate their use of motivational interviewing to address any barriers to completing the screening that may be verbalized by the participant. In weeks 4–6 of the intervention, participants receive one motivational text message per week to encourage them to complete their screening. The three text messages were among those most preferred by focus groups during the project’s learner verification phase. The intervention is completed in 6 weeks or potentially earlier if the participant reports completion of their FIT.

The educational tri-fold brochure provided to all participants was professionally designed and incorporated focus group input through learner verification. The brochure cover has the TUNE-UP header and is subtitled, *What Black Men and Women need to know about Colorectal Cancer Screening*, and features a photo of a middle-age African American couple. The brochure’s inner panels include information on the different CRC screening tests, emphasizing the stool-based test; an anatomical picture of the human colon with polyps; lists of modifiable and nonmodifiable CRC risk factors; questions/answers about CRC screening and the colonoscopy procedure; and a short testimonial about overcoming the fear of completing CRC screening. The back cover provides information about covering the cost of CRC screening, sources of additional information including the NCI and the American Cancer Society, and TUNE-UP contact information.

## Data collection and measures

### Overview

**Fig. 2:**
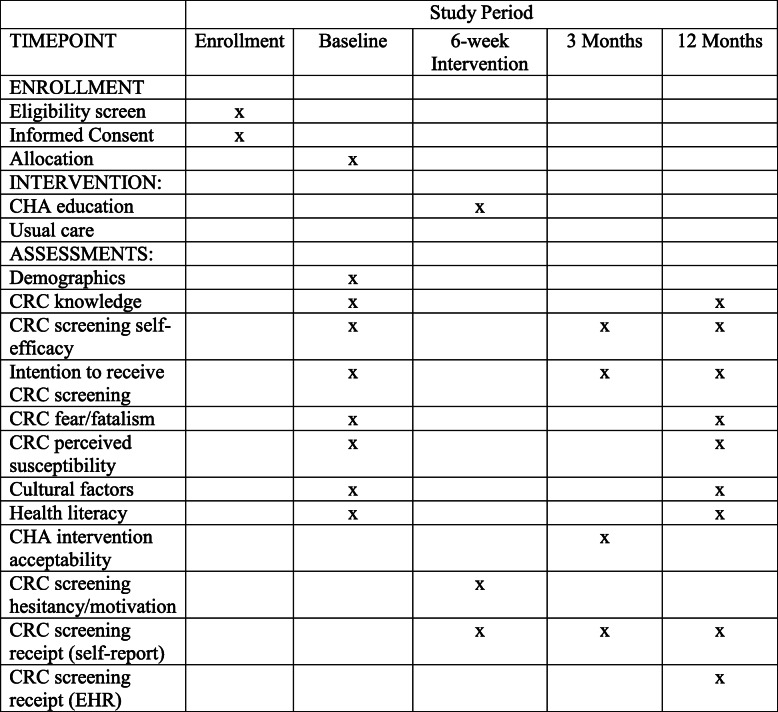
Schedule of enrollment, interventions, and assessments

The data collection occurs over three time periods: baseline, 3 months after baseline, and 9–12 months after baseline (Fig. 2). The surveys are administered in English only to African American research participants.

### Primary outcome

The primary outcome is the completion of the stool-based CRC screening within 12 months after the baseline survey completion. In the 3-month and 12-month follow-up surveys, the CRC screening measure asks about colonoscopy and stool blood tests. These tests are described prior to questions about each test and testing frequency [23]. Completion is determined by self-report in the follow-up survey with additional confirmation by reporting of FIT completion in partnership with collaborating CHCs.

### Secondary outcomes and other measures

The baseline survey collects patient information on demographics, insurance status, health literacy, communication with health professionals, trust in doctors, overall health status, and tobacco use. In addition, there are questions on CRC knowledge, CRC screening self-efficacy, CRC fear and fatalism, CRC perceived susceptibility, cultural factors using the multi-construct African American Cultural Survey, beliefs about cancer and CRC, and CRC screening intentions using validated measures [21]. The secondary outcomes to be measured at 12 months are CRC knowledge and CRC screening self-efficacy. At the 3-month follow-up survey, data are collected on receipt of CRC screening, CRC self-efficacy, CRC screening intentions, and intervention acceptability for intervention arm participants. At the 12-month follow-up survey, patients will respond again to questions on receipt of CRC screening, communication with health professionals, and trust in doctors. In addition, patients will respond to questions on knowledge, self-efficacy, fear and fatalism, perceived susceptibility, cultural factors using the multi-construct African American Cultural Survey, beliefs about cancer and CRC, and CRC screening intentions.

### Sample size calculation

Study sample size and power considerations are based on the primary outcome of receiving stool-based CRC screening. Based on a systematic review, we estimate a 20% increase in CRC screening among intervention arm participants and a 5% increase among control arm participants [24]. The larger the observed effect between arms, the lower the number of participants required to detect a significant difference at *α* = 0.05 and power of 80%. The total sample size, taking account of 40% attrition, was calculated to be 244 participants (122 per treatment arm).

### Patient recruitment challenges and solutions

The novel coronavirus pandemic and attendant prioritization of COVID-19 testing and vaccination by partnering CHCs exacerbated CHC staffing challenges and made it more difficult for CHCs to fulfill essential recruitment tasks, in particular, monthly dissemination of recruitment messaging through CHCs’ electronic health record systems. Once virtual and in-person meetings with CHC leadership teams were scheduled however, workable solutions were identified with each CHC regarding identifying the project coordinator’s CHC contacts and establishing mutual understanding about CHCs’ dissemination of email/text/phone messaging to potentially eligible patients to contact the study coordinator for study eligibility determination. Phone contacts are repeated as necessary to enroll participants. The eligibility criteria exclude those who are up to date with USPSTF CRC screening guidelines, so there are challenges to identify those patients due for screening. Also, participants from the same household were excluded to avoid possible study contamination. The research team developed two versions of a recruitment flyer, one for posting within partnering CHCs and the other for community posting/dissemination. It was important to ensure that both CHCs had an adequate supply of flyers for posting in waiting rooms and patient exam rooms and to encourage CHC medical staff to discuss study participation with clients during in-person visits. Additional participant recruitment methods included participant monetary incentives for baseline and follow-up survey completion, promotion by collaborating African American churches, regular broadcasting of a radio advertisement during programming with significant study population listenership, engaging potential participants at community events (e.g., food distribution), and flyers posted at local businesses (e.g., barbershops and beauty salons). There are no special criteria for discontinuing or modifying allocated interventions; however, participants may withdraw from the study at any time.

### Fidelity

To achieve fidelity of the CHA-delivered educational intervention, the CHA training provided by the research team informed the CHA about the essential concepts and significance of—and ways to maintain—fidelity. Maintaining intervention fidelity was presented in the context of essential skills and responsibilities of the CHA. Fidelity checklists were created to guide the CHA through each participant encounter and facilitate the CHA’s documentation of pertinent information from the encounter. These included a checklist for the in-person educational presentation and for each of two follow-up phone calls made by the CHA. The fidelity checklists, especially those for the follow-up phone calls, also served as conversation scripts for the CHA and were designed to facilitate the use of motivational interviewing. Training of the CHA culminated with the CHA practicing delivery of the scripts with research team members to simulate CHA-client interactions. Implementing an educational intervention with CHA counseling support or phone contact or usual educational materials about CRC screening plus a resource list will not affect any other concomitant care during the trial.

### Data management

Baseline and 12-month follow-up surveys are completed face-to-face and 3-month follow-up surveys are administered over the phone using paper surveys. Participants are identified by an ID number. A separate file is maintained with matched ID numbers and contact information to facilitate follow-up assessment administration. Date management is facilitated using the REDCap research management software platform [25]. REDCap is a secure Web application for building and managing surveys and databases and provides data export to statistical packages. Paper surveys are directly entered into REDCap and then exported to a password-protected network drive where SAS will be used for cleaning, coding, labeling, and statistical analysis. Hard copy data will be stored in a locked office accessible only by authorized study team members. There are no formal stopping rules for the trial since there are no anticipated problems expected to be detrimental to the participant. In addition, there are no anticipated adverse events and harms from the educational screening intervention.

### Statistical analysis

All statistical analyses will be performed using SAS v. 9.4. First, summary statistics will be generated for all variables. Then, bivariate analyses will be conducted for key variables to compare differences between the intervention and control arms. Categorical variables will be compared using chi-square tests; continuous variables will be compared using two-sample *t*-tests. The multivariate analyses will be conducted using the GEE procedure in SAS that implements the generalized estimating equations regression. Analyses will be performed using an intent-to-treat approach that includes all participants randomized. Significance tests will be two-sided and employ an overall significance level of *α* = 0.05. Sensitivity analyses will be employed to assess the impact of any missing data. For each arm, the proportion of participants who receive CRC screening by 12 months will be computed. The chi-square test will be used to compare the two treatment arms for screening receipt. If the proportion of participants receiving CRC screening in the intervention arm is at least 15% higher than that in the control arm, the intervention will be determined to be effective. To assess the influence of key covariates (age, income, marital status, education, insurance status) on the primary outcome, regression analysis will be employed. In addition, to investigate treatment effects over time (baseline, 3 months, and 12 months), we will estimate hazard ratios by fitting Cox proportional models using time-varying covariates. In these models, the receipt of CRC screening will be regressed at each actual time point on time elapsed since baseline to test for an interaction between time and treatment group. The basic model will be repeated, adjusting for age, income, marital status, education, and any other significant covariates at baseline. To test the proportional hazard assumption, the Kaplan-Meier curve and the Schoenfeld residuals will be used for fixed and time-varying covariates, respectively.

**Fig. 3:**
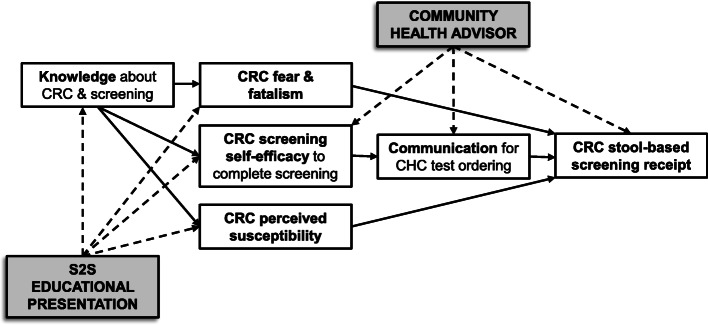
Conceptual model for TUNE-UP project

Mediation and moderation analyses will be conducted to determine why and when variables are related, respectively. We will focus on specific mediators to include CRC perceived susceptibility, CRC fear and fatalism, CRC self-efficacy, and CRC knowledge in the conceptual model (Fig. 3).

**Fig. 4:**
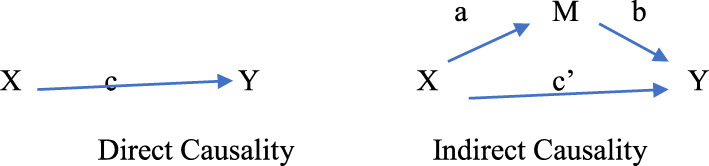
Path diagram for mediation models

The complexities of the relationships between the exogenous variables (*X*) and the endogenous variables (*Y*) as a function of the mediators (*M*_*n* = 1–4_) will be examined using four separate regression models. We will ensure that the assumptions of continuous measurements (i.e., that all variables are measured on a continuous scale), normality (i.e., normal distribution of all variables), independence (i.e., that there is no correlation of errors from one observation to another observation), and linearity (i.e., evidence linear relationships among the variables) are met. We will assess direct causality and indirect causality using standardized regression coefficients (i.e., betas) to determine the direction and magnitude of the effect of one variable upon the other variable (see Fig. 4).

We will confirm that the mediators (i.e., CRC perceived susceptibility, CRC fear and fatalism, CRC self-efficacy, and CRC knowledge) are caused by the exogenous variables *X* and are also the causes of the endogenous variables *Y*. We will also determine if *X* loses its significance when *M* is included in the model. Specifically, we will perform regression analyses and confirm the significance of the relationship between the exogenous (i.e., independent variable (IV)) and endogenous variables (i.e., dependent variable (DV)), the exogenous and mediator variable, and the mediator and the endogenous variables in the presence of the IV (i.e., *M*|*X* ➔ *Y*), and lastly, we will confirm the lack of significance (or the meaningful reduction in effect) of the relationship between *X* and *Y* in the presence of the mediators.

Additionally, we will assess the moderator variables (i.e., sex, health literacy, family history of cancer) to determine under what condition (i.e., when) a given predictor *X*/independent variable (IV) is related to an outcome *Y*/dependent variable (DV). We will assess whether the moderation effect is enhancing (i.e., that is, increasing the moderator would increase the effect of the predictor on the outcome), buffering (i.e., that is, increasing the moderator would decrease the effect of the predictor on the outcome), or antagonistic (i.e., where increasing the moderator would reverse the effect of the predictor on the outcome). We will apply hierarchical multiple regression to examine the effects of the moderating variable. We will assess the interaction effect between *X* and *M* and whether or not such an effect significantly predicts the occurrence of *Y*. All variables will be standardized to facilitate data interpretation and avoid multicollinearity. We will fit a regression model predicting the outcome from both the predictor and moderator variables. We will check that both the effect of the predictor and the effect of the moderator, as well as the model (*R*^2^), are statistically significant. Then, we will fit another regression model that includes the interaction effect of the first model and check for a statistically significant *R*^2^ change and a statistically significant effect of the interaction term to determine if moderation is occurring (i.e., both are statistically significant).

Mediation analysis allows assessment of each covariate’s impact on the primary outcome independent of whether the intervention’s effect is significant, thus adding to the scientific understanding of the relevance of each. To examine the mediating effects on the primary outcome of each of the short items scored on a Likert-type scale, we will follow the strategy of Baron and Kenny [26]. We will conclude that the candidate covariate is indeed a mediator of the effects of the intervention if (1) the treatment group indicator is significant and (2) the candidate covariate is significant, and the coefficient estimate of the treatment group indicator is smaller in absolute value than its counterpart in analysis.

To assess the impact of candidate moderators, we will use the Cox proportional hazard model and other appropriate survival analysis models to investigate the primary outcome on the treatment group, each candidate moderator, and the interaction of treatment and each candidate moderator at each measurement time point.

### Cost-effectiveness analysis

To accomplish the cost-effectiveness study objective, we will conduct a randomized controlled community trial-based cost-effectiveness analysis to determine whether CHA intervention is worth implementing compared to the standard of care from a payer perspective. The effectiveness of CHA intervention will be measured in terms of CRC screening rate, while costs will be measured using the micro-costing approach to capture resource utilization [27, 28]. The incremental cost-effectiveness ratio (ICER) will estimate the cost-effectiveness of the intervention compared to the standard of care. Deterministic sensitivity analysis will be carried out to identify the drivers of the ICER estimation. Additionally, we will estimate the 95% confidence intervals (CIs) for ICERs using a bootstrapping approach and to analyze the impact of uncertainty on variables and the sampling uncertainty (trial-based economic evaluation).

## Discussion

The TUNE-UP trial will aid in understanding how to improve stool-based CRC screening in an uninsured and underinsured African American patient population whose source of primary health care is through CHCs. While there have been several previous trials to test the effectiveness of clinic-based interventions to increase stool-based CRC screening in large public hospital or primary care settings, there have been very few studies conducted with CHCs focusing exclusively on an African American patient population, where CRC disparities are ongoing [8, 29]. The TUNE-UP trial utilizes evidence-based education materials combined with a CHA intervention outside of a hospital or cancer center setting, so the trial approximates pragmatic challenges encountered by CHCs to provide regular stool-based testing to their patients using an education and outreach strategy.

There are several ethical considerations involved in monitoring, consent, and dissemination provisions. The trial steering group meets weekly, and the IRB Ethics Committee meets annually to review conduct throughout the trial period for auditing trial conduct. The TUNE-UP study is IRB approved and subject to continuing IRB approval by the Florida A&M University IRB (IRB#1439452-2). Any important changes to the protocol will be communicated to the IRB and the funder. Next, a copy of the revised protocol would be documented and updated in the clinical trial registry. Because of the educational nature of the study, there is no anticipated harm and compensation for trial participation. The trial results will be reported to the clinical trial registry at the end of the study period and published in a peer-reviewed publication. The datasets analyzed during the current study and statistical code are available from the corresponding author on reasonable request, as is the full protocol.

Through the conceptual framework, the trial will test the relationships between several health behavior constructs to better understand the mechanisms by which the intervention affects beliefs and behaviors of patients to make the decision to participate in stool-based CRC screening. By identifying specific health behavior constructs of import to CRC screening, the research will identify areas of focus for future intervention studies to improve intervention components and increase CRC screening participation. There also may be a subset of patients who are uninformed about the opportunity to participate in CRC screening, and this trial may potentially reach these patients with low CRC knowledge and increase their knowledge, which may increase screening in this subgroup.

The addition of an exploratory aim on cost-effectiveness will add to the literature to approximate costs for the intervention to assist financially constrained CHCs to decide if hiring an additional staff member might improve stool-based test return rates and thus improve their cancer screening programs. In a CRC screening study conducted in El Paso, TX, the cost per additional person screened for their *promotora* intervention was $104 when compared to the comparison group [30]. By providing accurate cost estimates on aspects of a CHA program which includes transportation and time costs, CHCs will be better able to make budget estimates to implement such a program in their communities to focus on medically underserved patient populations and contribute to the effort of addressing cancer disparities. Over time, the CRC CHA position could be built into the health center operations to provide navigation and training for other screening activities or navigation for follow-up of positive screening tests.

## Trial status

The trial is in progress and enrolling participants. Trial enrollment began in April 2021 and will continue until March 2023.

## Data Availability

No data will be shared until the study is completed and will require a data sharing agreement.

## Abbreviations

ACS: American Cancer Society
CHA: Community health advisor
CHC: Community health center
CRC: Colorectal cancer
NCI: National Cancer Institute
S2S: Screen to Save
USPSTF: United States Preventive Services Task Force

## References

[1] Siegel RL, Miller KD, Fuchs HE, Jemal A (2021). Cancer statistics, 2021. CA Cancer J Clin..

[2] DeSantis CE, Miller KD, Goding Sauer A, Jemal A, Siegel RL (2019). Cancer statistics for African Americans, 2019. CA Cancer J Clin..

[3] Joseph DA, King JB, Dowling NF, Thomas CC, Richardson LC (2020). Vital signs: colorectal cancer screening test use - United States, 2018. MMWR Morb Mortal Wkly Rep..

[4] Carethers JM (2018). Clinical and genetic factors to inform reducing colorectal cancer disparities in African Americans. Front Oncol..

[5] DeGroff A, Coa K, Morrissey KG, Rohan E, Slotman B (2014). Key considerations in designing a patient navigation program for colorectal cancer screening. Health Promot Pract..

[6] Davidson KW, Barry MJ, Mangione CM, Cabana M, Caughey AB, U. S. Preventive Services Task Force (2021). Screening for colorectal cancer: U.S. Preventive Services Task Force Recommendation Statement. JAMA..

[7] Wolf AMD, Fontham ETH, Church TR, Flowers CR, Guerra CE, LaMonte SJ (2018). Colorectal cancer screening for average-risk adults: 2018 guideline update from the American Cancer Society. CA Cancer J Clin..

[8] Cusumano VT, May FP (2020). Making FIT count: maximizing appropriate use of the fecal immunochemical test for colorectal cancer screening programs. J Gen Intern Med..

[9] Gwede CK, Davis SN, Quinn GP, Koskan AM, Ealey J, Abdulla R (2013). Making it work: health care provider perspectives on strategies to increase colorectal cancer screening in federally qualified health centers. J Cancer Educ..

[10] The Lancet Gastroenterology and Hepatology (2021). USPSTF recommends expansion of colorectal cancer screening. Lancet Gastroenterol Hepatol.

[11] Lin JS, Perdue LA, Henrikson NB, Bean SI, Blasi PR (2021). Screening for colorectal cancer: updated evidence report and systematic review for the US Preventive Services Task Force. JAMA..

[12] Ebner DW, Kisiel JB (2020). Stool-based tests for colorectal cancer screening: performance benchmarks lead to high expected efficacy. Curr Gastroenterol Rep..

[13] Attipoe-Dorcoo S, Chattopadhyay SK, Verughese J, Ekwueme DU, Sabatino SA, Peng Y, Community Preventive Services Task Force (2021). Engaging community health workers to increase cancer screening: a community guide systematic economic review. Am J Prev Med..

[14] Escoffery C, Fernandez ME, Vernon SW, Liang S, Maxwell AE, Allen JD (2015). Patient navigation in a colorectal cancer screening program. J Public Health Manag Pract..

[15] Santos SL, Tagai EK, Scheirer MA, Bowie J, Haider M, Slade J (2017). Adoption, reach, and implementation of a cancer education intervention in African American churches. Implement Sci..

[16] National Ceancer Institute (2021). Screen to Save: NCI colorectal cancer outreach and screening initiative.

[17] Whitaker DE, Snyder FR, San Miguel-Majors SL, Bailey LO, Springfield SA (2020). Screen to Save: results from NCI’s colorectal cancer outreach and screening initiative to promote awareness and knowledge of colorectal cancer in racial/ethnic and rural populations. Cancer Epidemiol Biomarkers Prev..

[18] Boutsicaris AS, Fisher JL, Gray DM, Adeyanju T, Holland JS, Paskett ED (2021). Changes in colorectal cancer knowledge and screening intention among Ohio African American and Appalachian participants: the screen to save initiative. Cancer Causes Control..

[19] Glanz K, Bishop DB (2010). The role of behavioral science theory in development and implementation of public health interventions. Annu Rev Public Health..

[20] Kiviniemi MT, Bennett A, Zaiter M, Marshall JR (2011). Individual-level factors in colorectal cancer screening: a review of the literature on the relation of individual-level health behavior constructs and screening behavior. Psychooncology..

[21] Thompson VL, Bugbee A, Meriac JP, Harris JK (2013). The utility of cancer-related cultural constructs to understand colorectal cancer screening among African Americans. J Public Health Res..

[22] Guide to Community Preventive Services (2021). The community guide.

[23] Vernon SW, Meissner H, Klabunde C, Rimer BK, Ahnen DJ, Bastani R (2004). Measures for ascertaining use of colorectal cancer screening in behavioral, health services, and epidemiologic research. Cancer Epidemiol Biomarkers Prev..

[24] Naylor K, Ward J, Polite BN (2012). Interventions to improve care related to colorectal cancer among racial and ethnic minorities: a systematic review. J Gen Intern Med..

[25] Harris PA, Taylor R, Thielke R, Payne J, Gonzalez N, Conde JG (2009). Research electronic data capture (REDCap)-a metadata-driven methodology and workflow process for providing translational research informatics support. J Biomed Inform..

[26] Baron RM, Kenny DA (1986). The moderator-mediator variable distinction in social psychological research: conceptual, strategic, and statistical considerations. J Pers Soc Psychol..

[27] Drummond MF, Sculpher MJ, Torrance GW, O’Brien BJ, Stoddart GL (2015). Methods for the economic evaluation of health care programmes.

[28] Frick KD (2009). Microcosting quantity data collection methods. Med Care.

[29] Myers RE, Sifri R, Daskalakis C, DiCarlo M, Geethakumari PR, Cocroft J (2014). Increasing colon cancer screening in primary care among African Americans. J Natl Cancer Inst.

[30] Lairson DR, Kim J, Byrd T, Salaiz R, Shokar NK (2018). Cost-effectiveness of community interventions for colorectal cancer screening: low-income Hispanic population. Health Promot Pract..

